# Establishment and Mechanism Study of a Primary Ovarian Insufficiency Mouse Model Using Lipopolysaccharide

**DOI:** 10.1155/2021/1781532

**Published:** 2021-11-16

**Authors:** Si-Ji Lv, Shu-Hui Hou, Lei Gan, Jing Sun

**Affiliations:** ^1^Department of Gynaecology, Shanghai First Maternity and Infant Hospital, School of Medicine, Tongji University, Shanghai 200092, China; ^2^Department of Obstetrics and Gynecology, Shanghai Ninth People's Hospital, Shanghai Jiao Tong University School of Medicine, Shanghai, China

## Abstract

This study is aimed at establishing a lipopolysaccharide- (LPS-) induced primary ovarian insufficiency (POI) mouse model and investigating the underlying mechanism. C57BL/6N female mice were intraperitoneally injected with low-dose LPS (0.5 mg/kg) once daily for 14 days, high-dose LPS (2.5 mg/kg) twice weekly for 2 weeks, or cyclophosphamide (CTX; 150 mg/kg) once weekly for 2 weeks. Ovarian function was assessed by measuring the length of estrous cycle, the number of primordial follicles, and the levels of serum hormones. Expression and production of interleukin 1*β* (IL-1*β*) were determined to evaluate ovarian inflammation. Histopathological examination was performed to examine ovarian fibrosis. TUNEL assay was carried out to evaluate granulosa cell apoptosis. Western blotting was performed to measure the levels of inflammation-, fibrosis-, and apoptosis-related proteins in the mouse ovaries. Like CTX, both low- and high-dose LPS significantly impaired ovarian functions in mice, as evidenced by extended lengths of estrous cycles, reduced counts of primordial follicles, and alterations in the levels of serum hormones. Also, LPS promoted granulosa cell apoptosis and ovarian fibrosis in mice. However, LPS but not CTX promoted IL-1*β* expression and production in mice. Moreover, LPS but not CTX enhanced TLR, p-p65, p65, and MyD88 expression in mouse ovaries, suggesting that LPS differs from CTX in triggering ovarian inflammation. In general, continuous low-dose LPS stimulation was less potent than high-dose LPS to affect the ovarian functions. In conclusion, LPS may induce ovarian inflammation, fibrosis, and granulosa cell apoptosis and can be used to establish a POI model in mice.

## 1. Introduction

Primary ovarian insufficiency (POI) refers to the cessation of ovarian function before the age of 40 years, with an incidence of approximately 1% by the age of 40 years and 0.1% by the age of 30 years. POI is characterized by amenorrhoea, along with insufficient sex steroids such as estradiol (E2) and an elevated level of serum follicle-stimulating hormone (FSH) [1]. The underlying causes for POI include genetic abnormalities (such as Turner syndrome and X-monosomy), autoimmunity, impaired metabolism (such as 17-OH deficiency and classic galactosaemia), exposure to radiation or chemotherapy, infections, and environmental pollutants and toxins [2–6]. Since POI represents a major cause of female infertility [7, 8], it is important to elucidate the molecular mechanisms of POI which is helpful for the development of effective therapeutic strategies.

Owing to the similarity of the estrous cycle between female mice and humans, many studies have used POI mouse models to investigate the pathogenesis of POI and develop therapeutic modalities [9–11]. Currently, chemotherapeutic agents, such as cyclophosphamide (CTX) and cisplatin, are widely used to establish POI animal models due to their irreversible cytotoxicity toward ovaries, including destroying oocytes and arousing follicular depletion [12–14]. Although chemotherapy is one of the potential causes of POI, chemotherapeutic agent-induced POI may not simulate the pathophysiological conditions of POI resulting from genetic, autoimmune, metabolic, and infectious factors [15]. Thus, it is necessary to establish an animal model that may simulate the complex pathogenic mechanisms of POI.

Multiple mechanisms have been reported to contribute to the development of POI, including ovarian inflammation, granulosa cell apoptosis, and ovarian fibrosis [16–18]. A recent study has demonstrated that intraperitoneal (i.p.) injection of low-dose lipopolysaccharide (LPS; 0.5 mg/kg) once daily for 6 days markedly reduces the sizes of the ovaries and uteri while increasing the number of preantral and atypical follicles of C57BL/6J mice. In addition, LPS may increase the proinflammatory mediators in the mouse ovaries, such as Toll-like receptor 4 (TLR4), NOD-like receptor family pyrin domain-containing 3 (NLRP3), interleukin 1*β* (IL-1*β*), and IL-18. Moreover, NLRP3 activation promotes ovarian granulosa cell pyroptotic death and ovarian fibrosis. These findings suggest that LPS treatment may induce inflammation, granulosa cell death, and fibrosis in mouse ovaries, causing POI [19]. Thus, we hypothesized that LPS treatment can be used to establish a POI mouse model, which may be employed for the investigation of pathogenesis of POI and the development of therapeutic strategies.

To test our hypothesis, C57BL/6N mice were treated with low-dose LPS (i.p. 0.5 mg/kg) for 14 consecutive days, high-dose LPS (i.p. 2.5 mg/kg) twice weekly, or CTX (i.p. 150 mg/kg) once weekly for 2 weeks to induce POI. The ovarian function, inflammation, fibrosis, and granulosa cell apoptosis were evaluated and compared among groups. Our results suggest LPS stimulation as a new method to establish a POI mouse model.

## 2. Materials and Methods

### 2.1. Animals

The animal study was approved by the Ethics Committee of Tongji University (#TJBG03621101; Shanghai, China). All experiments were performed following the Guide for the Care and Use of Laboratory Animals of Tongji University. A total of 40 C57BL/6N female mice aged 6 to 8 weeks were purchased from Beijing Vital River Laboratory Animal Technology (Beijing, China) and housed in a temperature- and humidity-controlled environment at the Animal Research Center of Tongji University with a 12 h/12 h light-dark cycle and free access to food and water.

### 2.2. POI Mouse Model

Mice were randomly divided into control, low-dose LPS, high-dose LPS, and CTX groups (*n* = 10/group). To induce POI, mice were intraperitoneally injected with 0.5 mg/kg LPS (L2630; Sigma-Aldrich, St. Louis, MO, USA) once daily for 14 days, 2.5 mg/kg LPS twice weekly for 2 weeks, or 150 mg/kg CTX (C0768; Sigma-Aldrich) once weekly for 2 weeks. At 2 weeks after treatment, mice were anesthetized with 1% sodium pentobarbital via intraperitoneal injection, and blood samples were collected through cardiac puncture. Mice were then sacrificed using cervical dislocation. The ovaries were immediately collected and fixed with 4% paraformaldehyde for histological examinations or stored at -80°C until use.

### 2.3. Monitoring the Estrous Cycle

The phase of estrous cycle was determined by daily observations of vaginal epithelial cell cytology. Epithelial cells were collected daily by vaginal lavage and visualized using a Nikon Eclipse E100 light microscope (Nikon, Japan) under bright-field. Images were acquired at 100x magnification using a Nikon DS-U3 imaging system. Cell morphology was observed and assessed to determine the cycle phase (Supplementary Figure 1).

### 2.4. Histopathological Examinations

The ovary tissues were fixed in 4% paraformaldehyde overnight, dehydrated, embedded in paraffin, and cut into 4 *μ*m thick sections. The sections were dewaxed, rehydrated, and subjected to hematoxylin and eosin (H&E), immunohistochemical (IHC), or Masson staining following standard methods. H&E staining was performed for primordial follicle counting as previously described [20]. For each mouse, the primordial follicles in three consecutive sections were counted. For IHC staining, the sections were incubated with 0.01 M sodium citrate buffer for 20–30 min for antigen retrieval. The endogenous peroxidase was inactivated using 3% hydrogen peroxide. After blocking with 3% bovine serum albumin (BSA) for 30 min at room temperature, the section was incubated with primary anti-*α*-smooth muscle actin (*α*-SMA) antibody (Servicebio, Wuhan, Hubei, China) at 4°C overnight, followed by phosphate-buffered saline (PBS) rinses. After an incubation with horseradish peroxidase- (HRP-) labeled secondary antibody at room temperature for 50 min, the section was stained using a DAB detection kit (DAKO, Agilent Technologies, Santa Clara, CA, USA) following the manufacturer's protocol. Images were acquired using an XSP-C204 microscope (COIC, Chongqing, China). The results were assessed by two independent pathologists in a blind manner. The staining intensity was scored as previously described [21]. For Masson staining, the collagen fibers in ovarian tissue samples were stained using a Masson staining kit following the manufacturer's instructions (Guge Biotechnology, Wuhan, Hubei, China). The blue-stained collagen fibers were observed using an optical microscope (DS-U3; Nikon, Japan) at a magnification of 100x. The Masson-positive area was measured using ImageJ v1.8.0 (NIH, Bethesda, MD, USA).

### 2.5. TUNEL Assay

Ovarian granulosa cell apoptosis was examined using a TUNEL assay kit (Servicebio) following the manufacturer's instructions. Briefly, deparaffinized tissue sections were incubated with proteinase K (20 mg/mL; Guge Biotechnology) at 37°C for 20 min, followed by incubation with permeabilization buffer at room temperature for 20 min. After equilibration at room temperature for 10 min, sections were incubated with terminal deoxynucleotidyl transferase and deoxyuridine triphosphate at 37°C for 2 h in a moist chamber. Nuclei were counterstained with DAPI. Images were acquired using a Nikon Eclipse Ti-SR microscope.

### 2.6. Enzyme-Linked Immunosorbent Assay (ELISA)

The levels of serum anti-Müllerian hormone (AMH), E2, FSH, and IL-1*β* were measured using the corresponding ELISA kit (Elabscience, Wuhan, Hubei, China) following the manufacturer's instructions.

### 2.7. Quantitative Real-Time PCR (qRT-PCR)

Total RNA was isolated from ovarian tissue samples using Trizol (RNAiso Plus; Takara, Japan) according to the manufacturer's instructions. One microgram of RNA was reversely transcribed into cDNA using a PrimeScript™ RT reagent kit (Takara), according to the manufacturer's protocol. Amplification was performed using TB Green® Premix EX TaqTM II (Takara) and gene-specific primers (Table 1; Sangon, Shanghai, China) on a qRT-PCR device (QuantStudio5, Thermo Fisher Scientific, Waltham, MA, USA). GAPDH was used as an internal control. The relative expression of the genes was calculated using the 2^-*ΔΔ*CT^ method.

### 2.8. Western Blotting

The ground ovarian tissues were lysed using RIPA buffer (Biotechwell, Shanghai, China). Total proteins were isolated by centrifuging the lysates at 12,000 rpm for 5 min. The protein concentrations were determined using a bicinchoninic acid kit (Biotechwell). The protein samples were separated on 8–12% SDS-PAGE gels and then transferred to a polyvinylidene fluoride membrane. After incubation with 5% BSA at room temperature for 2 h, the membrane was incubated with primary antibody against TLR (1 : 1000; Cell Signaling Technology, Danvers, MA, USA), Bcl-2 (1 : 1000; Affinity, Scoresby, Victoria, Australia), collagen type I alpha 1 chain (Col1A1; 1 : 1000; Affinity), *α*-SMA (1 : 1500; Servicebio), Bax (1 : 1500; ImmunoWay Biotechnology, Plano, TX, USA), p-p65 (1 : 1000; ImmunoWay Biotechnology), MyD88 (1 : 1000; ImmunoWay Biotechnology), p65 (1 : 1000; Affinity), or GAPDH (1 : 2000; Biotechwell) overnight at 4°C. After incubation with HRP-conjugated secondary antibody (1 : 2000; Jackson Immuno, West Grove, PA, USA) at room temperature for 2 h, the protein bands were detected using an enhanced chemiluminescence kit (Biotechwell).

### 2.9. Statistical Analysis

Data were expressed as the mean ± standard error (SEM) of the mean. Statistical analysis was performed using SPSS 18.0 (IBM, Armonk, NY, USA). Differences between groups were compared using one-way analysis of variance, followed by Student's *t*-test. A value of *P* < 0.05 was considered statistically significant.

## 3. Results

### 3.1. LPS Stimulation Impairs Ovarian Function in Mice

**Figure 1:**
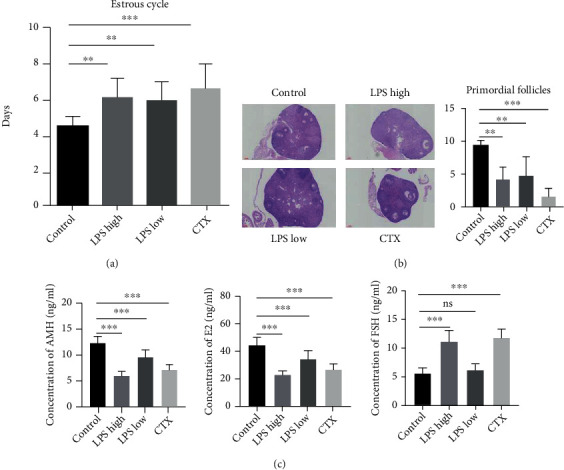
Lipopolysaccharide- (LPS-) induced ovarian dysfunction in mice. C57BL/6N female mice (6- to 8-week-old) were intraperitoneally injected with control, low-dose LPS (0.5 mg/kg once daily), high-dose LPS (2.5 mg/kg twice weekly), or cyclophosphamide (CTX; 150 mg/kg once weekly) for 2 weeks. (a) The lengths of estrous cycles were determined by daily observations of vaginal epithelial cell cytology. (b) Hematoxylin and eosin staining was performed to count primordial follicles (black arrow). Scale bar: 100 *μ*m. (c) Enzyme-linked immunosorbent assay (ELISA) was conducted to measure the levels of serum AMH, E2, and FSH at 2 weeks after treatment. Data are expressed as the mean ± standard error of the mean (SEM). ^∗∗^*P* < 0.01 and ^∗∗∗^*P* < 0.001; ns: nonsignificant; *n* = 10. AMH: anti-Müllerian hormone; E2: estradiol; FSH: follicle-stimulating hormone.

To investigate whether LPS treatment could induce POI in mice, the length of the estrous cycle, the count of primordial follicles, and the levels of serum pituitary/ovarian hormones were detected. As shown in Figure 1(a), like CTX, both low- and high-dose LPS significantly extended the length of estrous cycle in mice compared with the control group. Although less potent than CTX, both low- and high-dose LPS remarkably reduced the count of primordial follicles in mice compared with the control group (Figure 1(b)). Furthermore, both high-dose LPS and CTX markedly reduced the levels of serum AMH and E2 while elevating the serum FSH level in mice. Low-dose LPS was less potent than high-dose LPS in reducing the levels of serum AMH and E2 and had little influence on the serum FSH level in mice (Figure 1(c)). These changes are consistent with the characteristics of POI [1], suggesting that high-dose LPS twice weekly or low-dose LPS once daily for two weeks may induce POI in mice.

### 3.2. LPS Stimulation Induces Ovarian Inflammation in Mice

**Figure 2:**
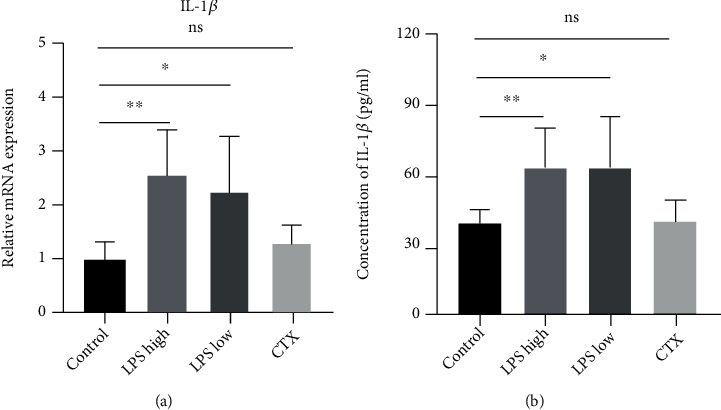
LPS stimulation promoted interleukin 1*β* (IL-1*β*) production in mice. Mice were sacrificed at 2 weeks after LPS or CTX treatment. (a) Quantitative real-time PCR (qRT-PCR) was performed to determine IL-1*β* mRNA expression in mouse ovary tissue samples. (b) ELISA was conducted to measure the serum IL-1*β* level in mice. Data are expressed as the mean ± SEM. ^∗^*P* < 0.05 and ^∗∗^*P* < 0.01; ns: nonsignificant, *n* = 10.

LPS is a potent inflammation trigger, and inflammation plays a critical role in the development of POI [16, 22, 23]. As shown in Figures 2(a) and 2(b), high- and low-dose LPS but not CTX significantly increased IL-1*β* production in mice, suggesting that LPS but not CTX induces ovarian inflammation in mice.

### 3.3. LPS Stimulation Induces Ovarian Fibrosis in Mice

**Figure 3:**
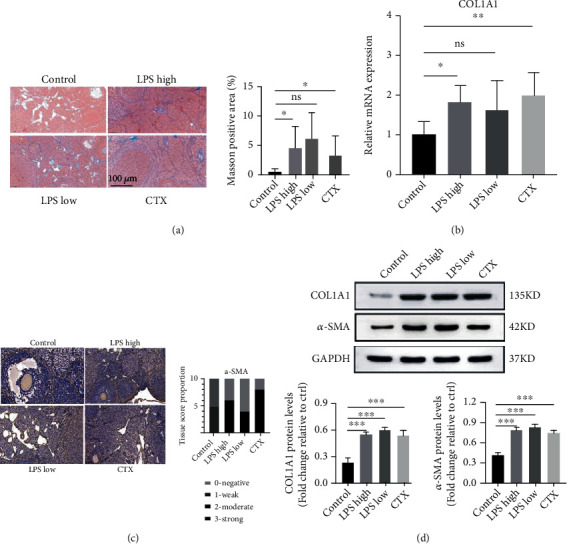
LPS stimulation induced ovarian fibrosis in mice. (a) Masson staining was performed to detect collagen deposition in mouse ovaries. Representative images are shown. Scale bar: 100 *μ*m. (b) qRT-PCR was performed to determine Col1A1 mRNA expression in the mouse ovaries. (c) Immunohistochemical staining was carried out to detect *α*-SMA expression in the mouse ovaries. Representative images are shown. Scale bar: 100 *μ*m. (d) Western blotting was performed to determine protein expression of Col1A1 and *α*-SMA in the mouse ovaries. Data are expressed as the mean ± SEM. ^∗^*P* < 0.05, ^∗∗^*P* < 0.01, and ^∗∗∗^*P* < 0.001, *n* = 10. Col1A1: type I procollagen; *α*-SMA: *α*-smooth muscle actin.

CTX stimulation causes ovarian fibrosis in mice [24]. Masson staining revealed that like CTX, high-dose but not low-dose LPS significantly increased collagen deposition in the mouse ovaries (Figure 3(a)). qRT-PCR revealed that high-dose but not low-dose LPS treatment significantly upregulated Col1A1 mRNA expression in the mouse ovaries (Figure 3(b)). IHC staining showed that like CTX, both high- and low-dose LPS stimulation enhanced *α*-SMA protein expression in the mouse ovaries (Figure 3(c)). Consistently, Western blotting showed that low-dose LPS, high-dose LPS, and CTX upregulated protein expression of Col1A1 and *α*-SMA in the mouse ovaries (Figure 3(d)). These findings suggest that like CTX, LPS induces interstitial fibrosis in the mouse ovaries.

### 3.4. LPS Stimulation Promotes Granulosa Cell Apoptosis in Mouse Ovaries

**Figure 4:**
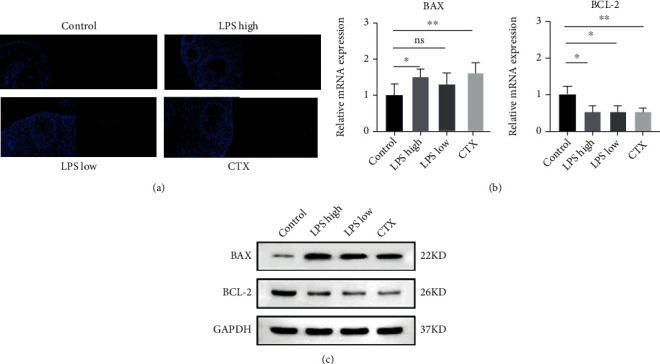
LPS stimulation induced granulosa cell apoptosis in mouse ovaries. (a) TUNEL assay was performed to examine granulosa cell apoptosis in the mouse ovaries. Representative images are shown, blue represents nuclear, and green represents apoptosis granule. Magnification 100x. (b) qRT-PCR and (c) Western blotting were performed to determine mRNA and protein expression of Bax and Bcl-2 in mouse ovaries. Data are expressed as the mean ± SEM. ^∗^*P* < 0.05 and ^∗∗^*P* < 0.01; ns: nonsignificant, *n* = 10.

Then, the cell apoptosis was evaluated in the mouse ovaries exposed to LPS treatment. TUNEL-positive granulosa cells were observed in the ovaries of LPS- and CTX-treated mice (Figure 4(a)). Moreover, LPS or CTX treatment significantly enhanced the mRNA and protein expression of Bax while attenuating Bcl-2 expression in the mouse ovaries (Figures 4(b) and 4(c)). These results suggest that like CTX, LPS treatment promotes granulosa cell apoptosis in the mouse ovaries.

### 3.5. LPS Activates TLR4/MyD88/NF-*κ*B Signaling in Mouse Ovaries

**Figure 5:**
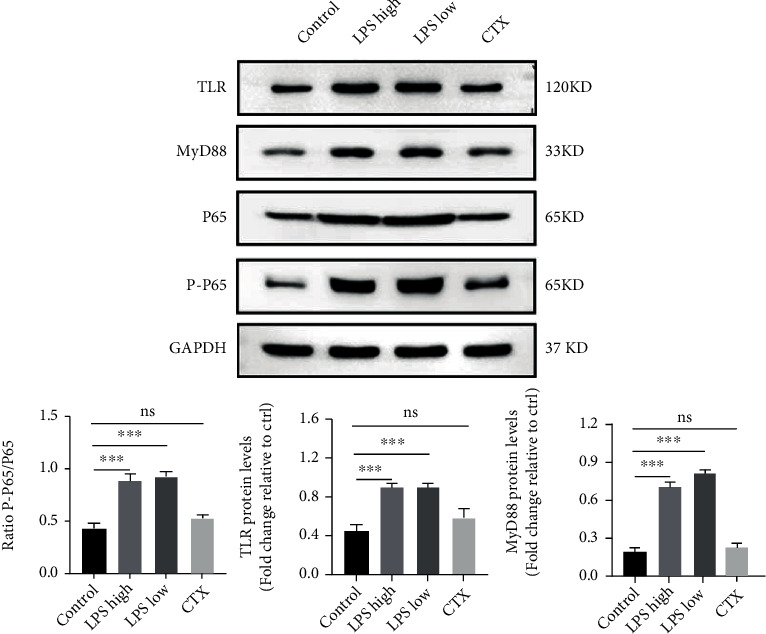
The expression of inflammation-related proteins. Western blotting was performed to determine protein expression of TLR, MyD88, p-p65, p-65, and GAPDH in the mouse ovaries. Data are expressed as the mean ± SEM. ^∗∗∗^*P* < 0.001; ns: nonsignificant.

The LPS/TLR4/MyD88/NF-*κ*B signaling pathway plays a critical role in the inflammation [25–27]. As shown in Figure 5, compared with the control group, both low- and high-dose LPS treatments significantly enhanced TLR, p-p65, p65, and MyD88 protein expression in the mouse ovaries. Conversely, CTX treatment attenuated the expression of these proteins compared with control. Taken together, these findings suggest that both LPS and CTX promote ovarian fibrosis and granulosa cell apoptosis and that LPS differs from CTX in inducing ovarian inflammation.

## 4. Discussion

POI has been a common disease and a major cause of female infertility in clinical practice. It has been reported that POI affects approximately 1% of women under 40 years of age, 0.1% under 30, and 0.01% of women under 20 years [1]. However, the effective treatments for POI are lacking, which may be partially ascribed to the poor understanding of the mechanisms underlying the pathogenesis of POI. A good animal model of POI should possess the characteristics of POI, which may be helpful for the investigation of potential mechanisms and treatments of POI.

Traditionally, CTX is employed for the establishment of the POI model due to its ovarian cytotoxicity [24, 28]. Although CTX has been shown to induce cortical fibrosis and follicle cell apoptosis that contribute to primordial follicle loss in the POI mice [24]. However, CTX stimulation is less associated with inflammation that, however, is an important cause of POI [23]. A recent study indicated that intraperitoneal low-dose LPS (0.5 mg/kg) once daily for 6 days was able to markedly reduce the sizes of the ovaries and uteri while increasing the number of preantral and atypical follicles of C57BL/6J mice, which was accompanied by the ovarian inflammation [19]. This study is aimed at investigating whether LPS treatment could induce POI in mice and the underlying mechanism was explored. Our results showed a 2-week treatment with 2.5 mg/kg LPS twice weekly or 0.5 mg/kg LPS once daily could induce POI in the mice. Like CTX, LPS stimulation resulted in the fibrosis and granulosa cell apoptosis in the mouse ovaries. Importantly, LPS but not CTX induced ovarian inflammation in the POI mouse model, as evidenced by the enhanced IL-1*β* production as well as the activation of the TLR/MyD88/NF-*κ*B pathway in the LPS-treated mice. These results suggest that in addition to ovarian fibrosis and follicle apoptosis, LPS also induces ovarian inflammation that is not observed after CTX stimulation.

Bromfield and Sheldon have reported that uterus or mammary gland infections with gram-negative bacteria cause infertility in cattle and that LPS exposure reduces the primordial follicle pool in the bovine ovarian cortex *in vitro* [29], suggesting that LPS may be used to establish POI animal models. After assessing the indicators of ovarian function, results showed that like CTX, both low- and high-dose LPS treatments significantly extended the length of estrous cycle, decreased the count of primordial follicles, reduced the levels of serum AMH and E2, and elevated serum FSH level in the LPS-treated mice, consistent with the characteristics of POI [1]. In addition, low-dose LPS was less potent than high-dose LPS regarding these effects. These data suggest that an LPS-induced POI mouse model is successfully established and high-dose LPS stimulation outperforms continuous low-dose LPS stimulation in inducing POI in mice. Wang et al. have treated C57BL/6N mice with 0.5 mg/kg LPS once daily for 6 days or 5 mg/kg LPS once daily for 2 days and found that low-dose LPS outperforms high-dose LPS in elevating the counts of preantral and atypical follicles and reducing the sizes of ovaries and uteri [19]. The different results between two studies are related to different LPS concentrations and treatment durations.

Inflammation is a major contributor to the pathogenesis of POI [22, 23]. Patients with POI have significantly increased systemic inflammation indicator neutrophil-lymphocyte ratio (NLR) compared with healthy controls, and the serum FSH level is positively related to NLR in patients with POI [23]. The anti-inflammatory drug can alleviate radiation-induced POI in rats by increasing AMH level and diminishing ovarian inflammation via inhibiting NF-*κ*B provoked inflammatory cytokines [30]. Our results showed that LPS stimulation significantly increased IL-1*β* mRNA expression and secretion as compared to controls. Consistently, Bromfield and Sheldon found that proinflammatory cytokines IL-1*β*, IL-6, and IL-8 accumulated in the supernatant of bovine ovarian cortex culture in an LPS concentration-dependent manner [29]. Wang et al. reported that serum IL-1*β* level dramatically increased after high-dose LPS treatment in mice. Moreover, both low- and high-dose LPS treatments elevate serum IL-6 levels and enhance IL-1*β* and IL-18 protein expression in the mouse ovaries [19]. These findings collectively indicate that LPS induces ovarian inflammation.

LPS activates the TLR4/NF-*κ*B signaling pathway to trigger inflammation [27, 31]. Wang et al. have shown that LPS treatment activates NF-*κ*B signaling and promotes cell apoptosis of bovine ovarian granulosa cells, as evidenced by significantly increased p-p65 and caspase-3 as well as Bax/Bcl-2 ratio [32]. In the present study, Western blotting revealed that LPS stimulation significantly enhanced protein expression of TLR4, MyD88, Bax, and molecules of NF-*κ*B signaling pathway while attenuating Bcl-2 expression in the mouse ovaries, further confirming that LPS activates the NF-*κ*B signaling pathway and induces cell apoptosis in the mouse ovaries. Importantly, the expression of inflammation-related proteins remained unchanged in the CTX-treated mice, suggesting that LPS but not CTX triggers ovarian inflammation in mice. Furthermore, LPS treatment exhibited similar or more potent effects on the upregulation of Col1A1 and *α*-SMA protein expression, suggesting that LPS is at least comparable to CTX in inducing ovarian fibrosis.

In this study, an LPS-induced POI mouse model is successfully established with LPS stimulation, and LPS not only induces fibrosis and granulosa cell apoptosis but also triggers inflammation that is not achieved by CTX stimulation in the mouse ovaries. These results suggest LPS stimulation may serve as a new strategy to establish POI mouse models for the investigation of the complex etiology of POI. Some studies have confirmed that the pathogenesis of endometriosis and polycystic ovarian syndrome is closely related to the inflammation [33, 34]. Thus, the use of LPS may be promising for the establishment of endometriosis and/or polycystic ovarian syndrome model.

## Figures and Tables

**Table 1:** Primers for quantitative real-time PCR.

Gene	Forward (5′–3′)	Reverse (5′–3′)
GAPDH	GACTGGATAAGCAGGGCGG	CCCAATACGGCCAAATCCGT
IL-1*β*	GAAATGCCACCTTTTGACAGTG	CTGGATGCTCTCATCAGGACA
COL1A1	TGACCTTCCTGCGCCTAATG	AAGTTCCGGTGTGACTCGTG
BAX	GGCCTTTTTGCTACAGGGTTTC	CAGCTTCTTGGTGGACGCAT
BCL-2	GCTGGGTAGGTGCATGTCTG	CAGGGGAGCAAAGCTACAAACT

## Data Availability

The data used to support the findings of this study are available from the corresponding author upon request.

## Supplementary Materials

Supplementary Materials Supplementary Figure 1: representative figures from vaginal lavages in 4 phases (proestrus, estrus, metestrus, and diestrus) of the estrous cycle in female rats ×100.
