# Supplementary material for: Establishment and Mechanism Study of a Primary Ovarian Insufficiency Mouse Model Using Lipopolysaccharide
Source: Anal Cell Pathol (Amst). 2021 Nov 16;2021:1781532. doi: 10.1155/2021/1781532 (PMC8610681; doi:10.1155/2021/1781532)

Supplementary Figure 1. Representative figures from vaginal lavages in 4 phases (proestrus, estrus, metestrus, and diestrus) of the estrous cycle in female rats×100.


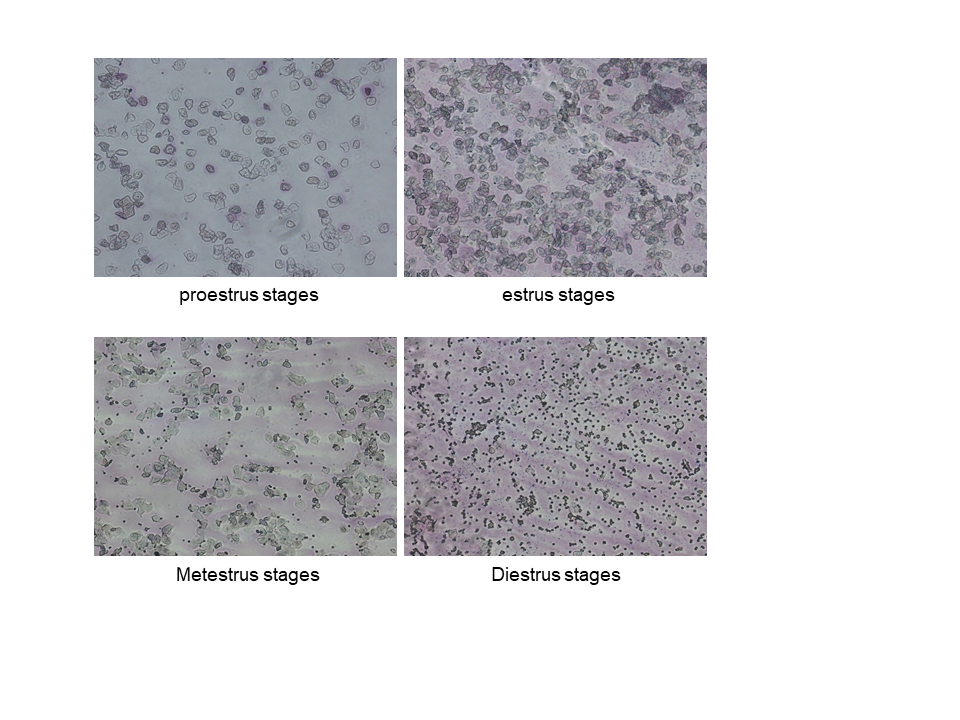

Supplement: Supplementary Materials — Supplementary Figure 1: representative figures from vaginal lavages in 4 phases (proestrus, estrus, metestrus, and diestrus) of the estrous cycle in female rats ×100. [file 1781532.f1.docx]
